# Bifunctional optogenetic switch for improving shikimic acid production in *E. coli*

**DOI:** 10.1186/s13068-022-02111-3

**Published:** 2022-02-07

**Authors:** Irene Komera, Cong Gao, Liang Guo, Guipeng Hu, Xiulai Chen, Liming Liu

**Affiliations:** 1 State Key Laboratory of Food Science and Technology, Jiangnan University, Wuxi, 214122 China; 2 International Joint Laboratory On Food Safety, Jiangnan University, Wuxi, 214122 China; 3 School of Pharmaceutical Science, Jiangnan University, Wuxi, 214122 China

**Keywords:** Metabolic regulation, Optogenetics, Protein degradation, Protein splitting, Shikimic acid

## Abstract

**Background:**

Biomass formation and product synthesis decoupling have been proven to be promising to increase the titer of desired value add products. Optogenetics provides a potential strategy to develop light-induced circuits that conditionally control metabolic flux redistribution for enhanced microbial production. However, the limited number of light-sensitive proteins available to date hinders the progress of light-controlled tools.

**Results:**

To address these issues, two optogenetic systems (TPRS and TPAS) were constructed by reprogramming the widely used repressor TetR and protease TEVp to expand the current optogenetic toolkit. By merging the two systems, a bifunctional optogenetic switch was constructed to enable orthogonally regulated gene transcription and protein accumulation. Application of this bifunctional switch to decouple biomass formation and shikimic acid biosynthesis allowed 35 g/L of shikimic acid production in a minimal medium from glucose, representing the highest titer reported to date by *E. coli* without the addition of any chemical inducers and expensive aromatic amino acids. This titer was further boosted to 76 g/L when using rich medium fermentation.

**Conclusion:**

The cost effective and light-controlled switch reported here provides important insights into environmentally friendly tools for metabolic pathway regulation and should be applicable to the production of other value-add chemicals.

**Supplementary Information:**

The online version contains supplementary material available at 10.1186/s13068-022-02111-3.

## Background

Precise metabolic flux regulation is a critical strategy to reprogram microbial cell factories to obtain high titer, yield, and productivity of various valuable compounds [1, 2]. To achieve metabolic regulation, both static regulation and dynamic system strategies are used to date. While some popular static regulation strategies, such as gene knockout [3], promoter engineering [4], RBS shuffling [5], etc., have been successfully used to redirect the carbon flux towards desired metabolites, they also cause cell growth defects by either blocking the synthesis of essential metabolites or accumulating toxic metabolites. To this end, dynamic regulation tools are needed to fine-tune cellular carbon flux without sacrificing cell growth [6].

Dynamic regulation tools represent a new frontier that enables controlling two or various competing pathways and subsequently channeling cell resources onto a targeted pathway [7]. So far, synthetic biology has contributed to the development of several regulatory strategies, including the TetR/LacI inverter-based toggle switch [8], temperature-induced dynamic system [9], RNAi based dynamic gene repressors [10], quorum sensing [11], and metabolite responsive circuits [12]. Optogenetics presents an attractive strategy and offers several advantages in dynamic control. First, optogenetics provides a low-cost approach compared to chemical inducers during scale-up fermentation [13]. Second, light is non-toxic to cellular metabolism and is compatible with any medium composition [14]. Third, light input can be turned on or off instantaneously, as it can be easily controlled with an electronic system to confer the desired signal intensity or frequency when needed [15]. Recently, an increasing number of optogenetic tools, such as OptoEXP [13], OptoINVRT7 [16], Opto-CRISPRi [14], eLightOn [17] and OptoLAC [18], have been constructed for various value-add chemical production processes. Yet, a limited number of photo-switchable proteins hinder the achievement of multifunctional and reversible light-inducible switches.

Regulatory switches are signal transduction systems capable of reversibly shifting between two states, allowing cells to sense and respond to specific signals for appropriate mechanism change [19]. Based on their regulation target, two types of regulatory switches, including transcription regulatory switches and post-transcriptional regulatory switches, were developed. Transcriptional switches are mainly designed by DNA binding proteins such as transcription factors, gene repressors (LacI, TetR, λCI), and transcription effectors (temperature, pH, and chemical inducers) [20]. Though the transcriptional level regulations are easy to control, they always cause delayed response time and leaky expression due to the already-translated target proteins. In contrast, post-transcriptional switches, including those based on protein and RNA interactions, allow quick and precise control. For example, the protein level regulation can interact directly with pathway enzymes abundance to manipulate cell function even at slow growth rates and low dilution [21]. These microbial protein switches can be designed in two approaches. On one hand, protein degradation could be achieved by adopting the native protein degradation system or the heterogenous proteasome by fusing different C-terminal degradation tags [22]. On the other hand, conditional protein degradation could be realized using various effectors (*potyvirus* protease, temperature, light, etc.) to trigger target protein degradation by adopting N-terminal degrons [23].

In the present study, we first report a strategy to expand optogenetic toolkits by reprograming the preexisting chemically induced repressor TetR and the tobacco etch virus protease (TEVp). Then, we engineered, optimized, and combined the dynamic units into a bifunctional optogenetic switch to simultaneously control gene expression and protein accumulation. Furthermore, this switch was used to decouple *E. coli* cell growth from the production pathway to improve shikimic acid titer up to 76 g/L, providing a promising way to construct economically attractive microbial cell factories.

## Results

### Design and characterization of the light-inducible transcription regulation unit

**Fig. 1:**
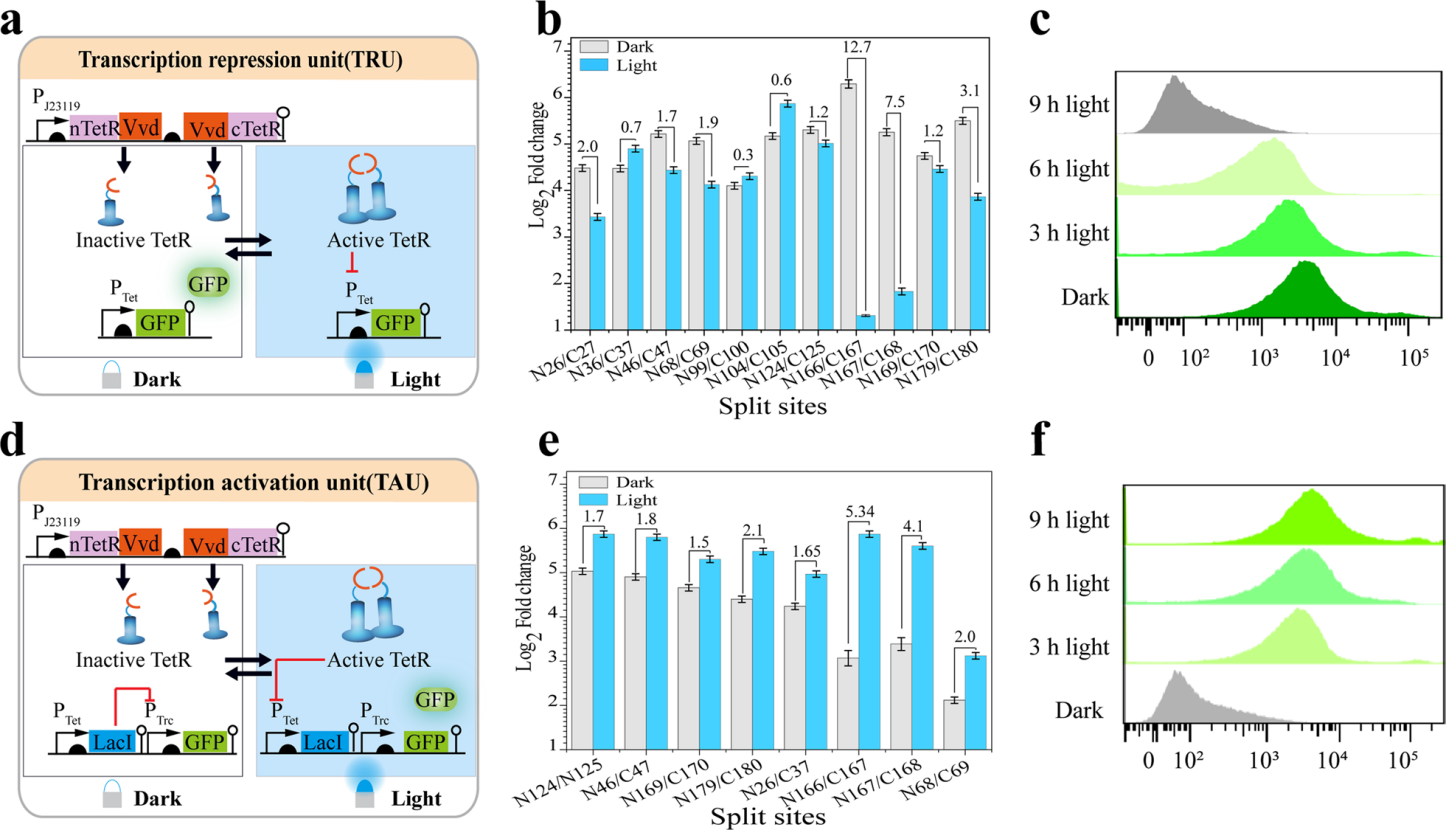
Design and assessment of the transcription regulation unit. **a** Design of the transcription repression unit. **b** Evaluation of the ability of selected splits to repress the transcription**. c** Flow cytometry for the 166 split-site under the transcription repression unit. (Grey: control no GFP; green: 166 split-site under the light condition and dark green: represent the 166 split-site under dark state. **d** Design of the transcription activation unit. **e** Evaluation of the transcription activation unit. **f** Flow cytometry for 166 split-site under the transcription activation unit. (Grey: control no GFP; green: 166 split-site under the light condition and dark green: represent the 166 split-site under dark state). The numbers above the bars indicate the fold change, calculated from the fluorescence ratio between the dark and the light phases

To assemble a bifunctional metabolic flux control tool, two units, the transcription activation unit and the transcription repression unit were constructed by mining the TetR repressor (Fig. 1). Moreover, to achieve light-induced recombination and splitting, blue light-sensitive VVD dimers[24] were fused to eleven TetR splits (26, 36, 46, 68, 99, 104, 124, 166, 167,169, and 179), which were designed using computer-based simulations and protein homology modeling according to the TetR crystal structure (PDB: 4V2G) (Fig. 1a, Additional file 1: Fig. S1). Specifically, the transcription repression unit was designed as a single layer circuit with a P*tet* promoter controlled by its cognate light-activated TetR repressor and an output green fluorescent reporter protein. In contrast, the transcription activation unit was designed as a double layered circuit in which an additional P*trc* promoter and *LacI* repressor were introduced on the transcription repression unit (Fig. 1d).

To test if the light-controlled TetR splits can induce the repression of the reporter gene, the fluorescence intensity of engineered strains containing the TetR-split plasmids was measured in the dark or illuminated states. The transcription repression unit results highlighted eight split pairs (S26, S46, S68, S124, S166, S167, S169, and S179) exhibiting functional recombination, repressing the fluorescence intensity ranging from 1.2 to 12.4-fold, respectively. The splits that exhibited the activation fold below one unit were classified inactive. Among them, the TetRN166/TetRC167 split pair showed the highest recombination efficiency and was thus selected for further studies (Fig. 1b). In contrast, the transcription activation unit performance was tested using each of the above eight functional split pairs, which was co-expressed with the transcription activation-containing plasmid. Compared to the control strain harboring a complete TetR, all the split pairs exhibited functional recombination with activation fluorescence ranging from 1.7-fold to 5.38-fold, respectively (Fig. 1e). To solve the crosstalk effect that may arise from genomic LacI, the optimal TetRN166/TetRC167 split pair was tested in a LacI knockout *E. coli* MG1655 strain, leading to a further increase in fluorescence activation range from 5.3-fold to 10.5-fold (Additional file 1: Fig. S2). These results demonstrated that the functional splits could be used in various functions depending on the tightness of the system required.

To characterize the performance of the transcription regulatory units, the tunability, system homogeneity, and cytotoxicity effects on the cells equipped with different units were examined. For the tunability, flow cytometry results revealed that tunable gene transcription activation or repression could be achieved by controlling cell exposure times to blue light (Fig. 1c, f). Next, the cell growth curve results showed that both units were noninvasive with negligible cytotoxicity on cell growth (Additional file 1: Fig. S3). Moreover, we characterized the light-inducible units. The results of single-cell fluorescence microscopy images demonstrated good population homogeneity (Additional file 1: Fig. S4).

### Design and optimization of the light-inducible protein accumulation unit

**Fig. 2:**
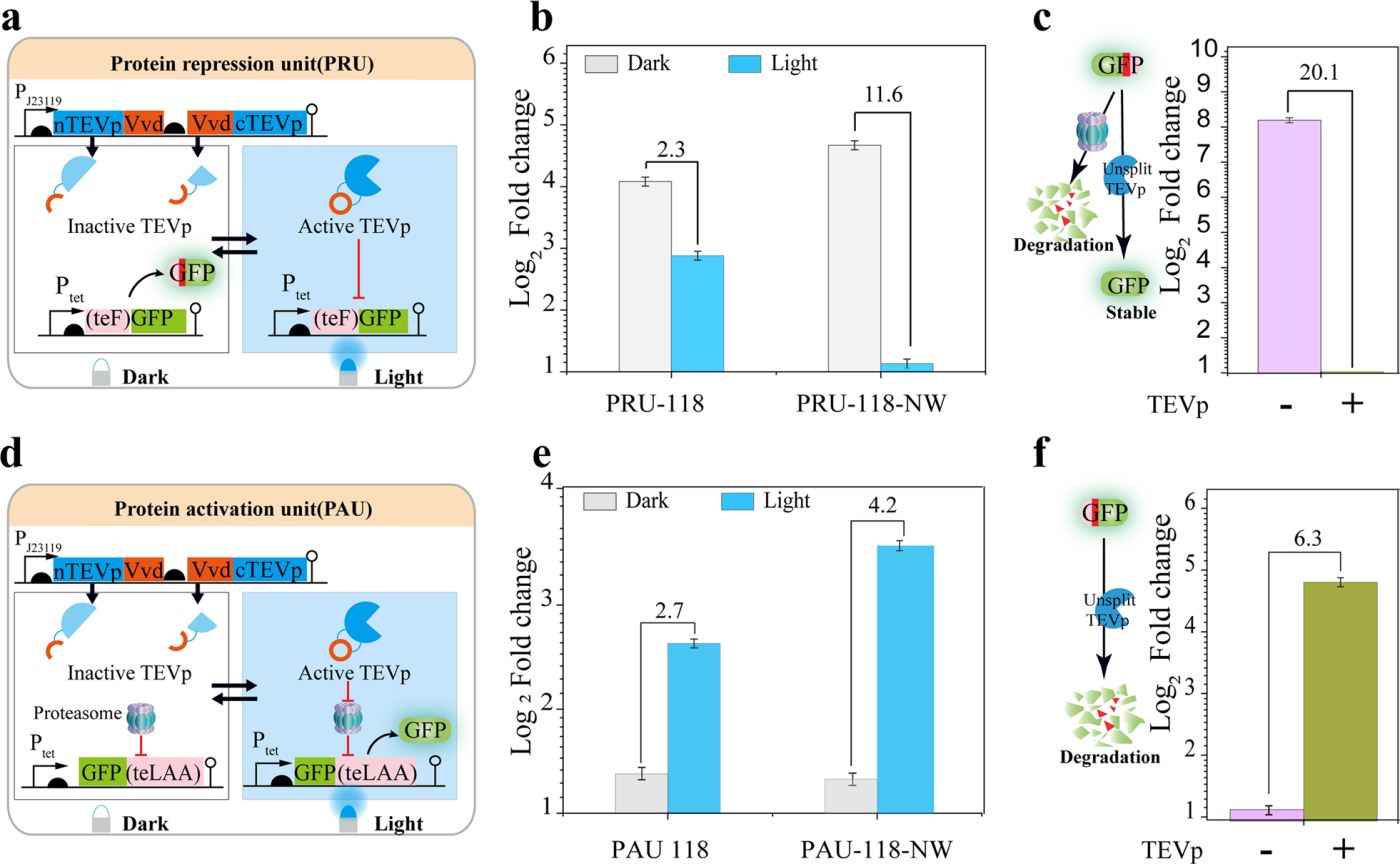
Design and evaluation of the protein regulation unit. **a** Design of the protein accumulation repression unit. **b** Assessment of the protein repression unit **c** Evaluation of the control design for the protein repression unit using unsplit TEVp. **d** Design of the protein accumulation activation unit. **e** Evaluation of the protein activation unit. **f** Assessment of the control design of the protein activation using unsplit TEVp on (PAU). The numbers above the bars indicate the fold change, calculated from the fluorescence ratio between the dark and the enlighten phases

Protein level regulation could provide more precise control of the target gene than transcriptional regulation. To rationally control protein accumulation, two light-inducible units (protein accumulation repression unit and protein accumulation activation unit) were designed. A pair of VVD-fused TEVp split was used to achieve light-induced protein degradation (Additional file 1: Fig. S5). Specifically, a cryptic N-terminal degron was fused to the GFP reporter protein in the protein accumulation repression unit. Consequently, upon the blue light-ON state, the activated TEVp could expose the N-degron of GFP and drive its degradation (Fig. 2a). In contrast, a C-terminal degron was tagged to the GFP reporter protein in the protein accumulation activation unit. As a result, upon the blue light-ON state, the reconstituted TEVp could precisely remove the C-terminal degron of GFP and protect it from degradation (Fig. 2d).

Having provided the VVD fusing position on the TEVp split fragment, we investigated if the VVD position could affect the recombination efficiency. Hence, two types of designs termed PRU-118-NW and PRU-118 were built by fusing the VVD to the C/C-ends and N/C-ends of the TEVp fragments, respectively (Fig. 2b). The efficiency of recombination was determined by comparing the fluorescence intensity of samples in the dark and light states. Results showed that the PRU-118-NW strain exhibited a mild leaky expression with 11.6-fold repression compared to the PRU-118 strain with 2.3-fold repression (Fig. 2b). Similarly, to provide the protein activation module, two types of protein accumulation activation units, terming PAU-118-NW and PAU-118, were built by fusing the VVD to the C/C-ends and N/C-ends TEVp fragments, respectively (Fig. 2d). Compared to the control strain with a complete TEVp (Fig. 2f), both strains harboring PAU-118 and PAU-118-NW could activate the accumulation of degron-tagged GFP through cleavage. The PAU-118-NW design displayed a 4.2-fold activation, making it 1.5-fold higher than the PAU-118 design (Fig. 2e).

To characterize the protein regulation tools, cells containing the tools were used to analyze the protein level tool tunability, homogeneity, and cytotoxicity. First, different activation or repression folds could be detected with various blue light intensities indicated that both units were tunable (Additional file 1: Fig. S6). In addition, the metabolic burden that the protein regulation tools impose on cellular metabolism and growth was investigated. Cell growth curves reveal that both units do not weaken cell growth (Additional file 1: Fig. S7). Furthermore, single-cell fluorescence images in PAU-118-NW and PRU-118-NW showed that the protein accumulation repression unit had better homogeneity than the protein accumulation activation (Additional file 1: Fig. S8).

### Design and characterization of the bidirectional switch

**Fig. 3:**
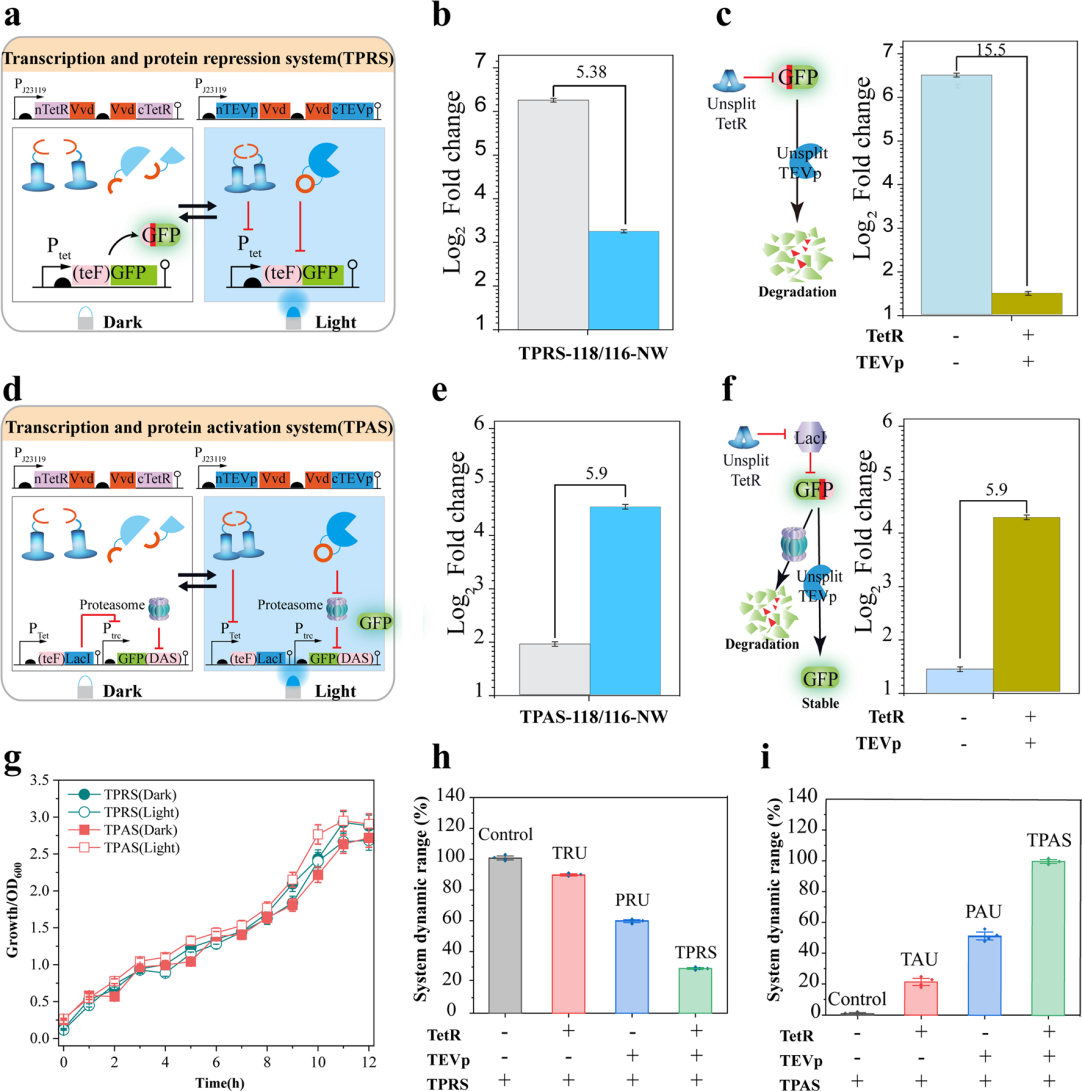
Design and evaluation of the transcription and protein regulation system. **a** Design of the transcription and protein repression system **b** Evaluation of the integrated TPRS. **c** Evaluation of the control design of the transcription and protein repression system. **d** Design of the transcription and protein activation system. **e** Evaluation of the TPAS. **f** Evaluation of the (TPAS) control design **g** Growth profile of the TPRS and TPAS containing strains under dark and light conditions. **h**, **i** Evaluation of the TPRS and TPAS component performance: **h** Assessment of the benefits of TPRS, transcription, and protein repression system and its modular components PRU, protein repression unit; and TRU, transcription repression units. **i** Assessment of the TPAS, transcription, and protein activation system, together with the components; PAU, protein activation unit; TAU, transcription activation units

Considering the potential to tune gene expression through transcriptional level regulation and control translated protein abundance by protein level regulation, the above two regulatory units were combined to establish a bilayered switch. The first layer consists of the transcription and protein repression system (TPRS) design, in which light-inducible TetR and TEVp were co-expressed with an N-terminal degron fused GFP (Fig. 3a). Therefore, when system input was switched from dark to light, the transcription activity of P*tet* promoter was repressed by the reconstituted TetR, and the translated GFP could be degraded by the reconstituted TEVp, leading to an output from GFP accumulation to repression (Fig. 3a). The second layer is the transcription and protein repression system (TPAS) design; an N-terminal degron fused LacI repressor was introduced into a P*trc* controlled C-terminal degron fused GFP. As a result, when system input was switched from dark to light, the abundance of LacI could be decreased at both transcriptional and protein levels to de-repress the expression and accumulation of GFP, leading to an output from GFP accumulation repression (Fig. 3d).

The performance of each system was evaluated by recording cell fluorescence intensity either in the dark or light-exposed states. In addition, two strains with complete TEVp and TetR expression were designed as controls (Fig. 3c, f). TPRS-118/166-NW and TPAS-118/166-NW showed 4.6-fold repression and sevenfold activation, respectively (Fig. 3b, e). The TPAS cells revealed a minimal GFP expression under the dark condition, representing 0.5-fold lower than the protein accumulation repression unit (Fig. 2e). This result indicated that tight control could be realized by integrating the double Layer regulatory systems (Fig. 3e).

To assess systems performance, the cytotoxicity, composability, homogeneity, and responsiveness of the two layers TPRS and TPAS, were evaluated. As shown in Fig. 3g, similar cell growth curves could be observed under the dark state and light state (OD = 2.8 for TPRS, OD = 2.75 vs. OD = 2.60 for TPAS, under dark and light conditions), indicating that both systems procure minimal to negligible cell growth. To assess the benefits of the bifunctional switch and its composability efficiency, we prepared four strains for each of the two systems, each harboring different functional components and a control strain carrying the output module deprived of any of the control switches. The assembly of units improved the tool performance; for example, TPRS provides 4.5-fold and threefold quicker control of gene and protein repression than transcription repression unit and protein repression unit. TPAS, on the other hand, controls gene and protein repression 5 to 1.6 times quicker than the transcription activation unit and protein activation unit, respectively (Fig. 3h, i). In addition, switch kinetic experiments were determined through a time lapse comparison of the systems to control the reporter gene accumulation or depletion. Results revealed that the equipment of both the transcriptional regulation system and the protein level regulation could endow strains with a twofold quicker response time (less than 30 min) and a 2.5-fold degradation rate (1800 fluorescent units per hour) than that of a single transcriptional level regulation (Additional file 1: Fig. S9). Furthermore, single-cell fluorescent microscopy images of both TPAS and TPRS showed good homogeneity with a less wide dynamic range (Additional file 1: Fig. S10).

### Light-inducible regulation of shikimic acid bioproduction

**Fig. 4:**
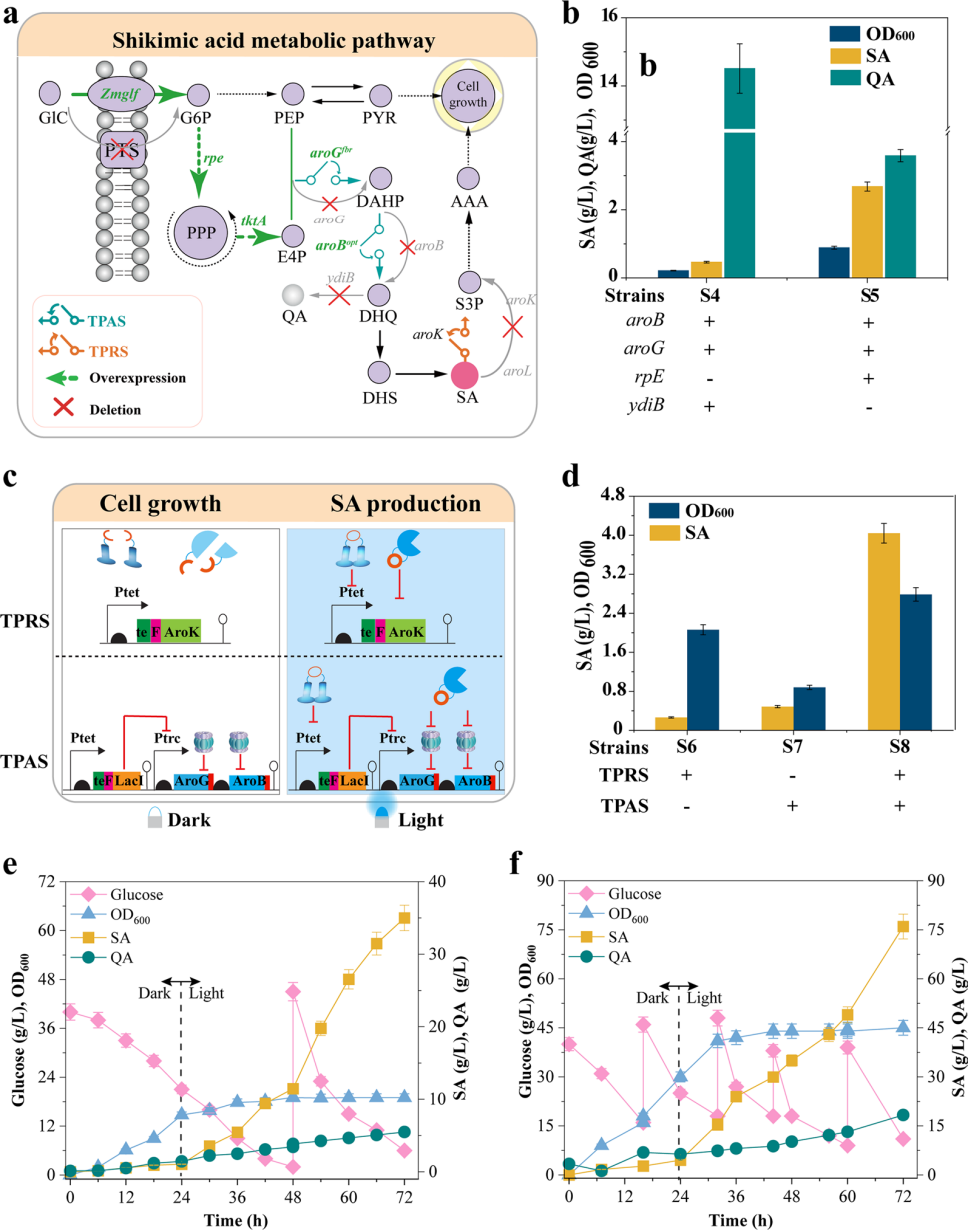
Shikimic acid production. **a** Light controlled shikimic acid production pathway. Light controlled shikimic acid production pathway. GlC, glucose; *Zmglf*, glucose facilitator from *Z. mobilis*; PTS, phosphotransferase system; G6P, glucose 6-phosphate; Ru5P, ribulose-5-phosphate; PEP, phosphoenolpyruvate; PYR, pyruvate; *aroB*^*opt*^, codons optimized DHQ synthase; *ydiB*, quinate/shikimate dehydrogenase; *aroG*^*fbr*^, feedback-resistant mutants of DAHP synthase; E4P, erythrose-4-phosphate; *aroK*, shikimate kinase I; DAHP, 3-deoxy-D-arabino-heptulosonate-7-phosphate; QA, Quinic acid; DHQ, 3-dehydroquinic acid; DHS, 3-dehydroshikimic acid; SA, shikimate; S3P, shikimate-3-phosphate; AAA, aromatic amino acids. Gene knockouts were presented in grey color. **b** Screening the suitable chassis strain for shikimic acid production in a rich medium. **c** Design of TPAS and TPRS for shikimic acid production. **d** Evaluation of bifunctional optogenetic switch (S8) and individual components, TPRS (S6) and TPAS (S7) systems, for shikimic acid production. **e** Fed-batch fermentation using NBS minimal medium in a 5 L bioreactor. **f** Fed-batch fermentation using an enriched medium in a 5 L bioreactor. Values are shown as the mean ± s.d. (*n* = 3)

Shikimic acid is an essential precursor for synthesizing oseltamivir, an antiviral agent used against H5N1 influenza [25]. Unless expensive AAA were supplemented into the minimal medium, a simple inactivation of shikimic acid depletion would cause cell growth defects [26, 27] (Fig. 4a). To solve this issue, the established bidirectional regulation switch was implemented to improve shikimic acid production without any chemical inducers or AAA supplementation in the growth medium. Thus, TPRS was designed to control cell growth, whereas TPAS was designed to direct the carbon flux towards the shikimic acid production when switched from dark to illuminated states (Fig. 4b).

To obtain a suitable strain for shikimic acid production, a previously engineered *E. coli* S4 was used as a chassis. This strain exhibited growth defects in the NBS minimal medium; it was first evaluated in minimal medium supplemented with 5 g/L tryptone for shikimic acid production. A total of 0.5 g/L of shikimic acid was produced after 72 h of fermentation, with 14.2 g/L of quinic acid detected. To decrease the accumulation of byproducts as well as improve shikimic acid production, an engineered strain of *E. coli* S5 with deletion of *ydiB* (encoding quinate/shikimic acid dehydrogenase) and overexpression of *rpE* (encoding Ru-5P 3-epimerase) was constructed and exhibited a shikimic acid titer increase of 5.4-fold (2.7 g/L) and a fourfold titer decrease of quinic acid (3.5 g/L) (Fig. 4b). Thus, *E. coli* S5 was selected for optogenetic systems verification in the NBS minimal medium.

To show the relationship between bidirectional regulation systems and titer improvement, three engineered strains were constructed, in which *E. coli* S6 harbors only TPAS, *E. coli* S7 harbors only TPRS, and *E. coli* S8 harbors both TPAS and TPRS (Fig. 4c). Fermentation results showed that *E. coli* S8 produced 4.20 g/L shikimic acid from 20 g/L glucose, while *E. coli* S6 and S7 could produce 0.46 g/L and 0.81 g/L shikimic acid (Fig. 4d). To further reveal the effects of fermentation conditions on shikimic acid production, fermentation temperature (Additional file 1: Fig. S11), glucose concentrations (Additional file 1: Fig. S12), and light switch times (Additional file 1: Fig. S13) were investigated. Results showed that the optimum conditions for temperature, glucose concentration, and light switch time were at 37 °C, 40 g/L, and 24 h, producing the highest shikimic acid titer of 4.6 g/L in shaker flasks with a yield of 0.07 g/g glucose and a productivity of 0.12 g/h·L (Additional file 1: Fig. S13).

Under the optimized conditions, strain S8 was cultured for 72 h in the NBS minimal medium using a 5 L fermenter (Fig. 4e). It produced 35 g/L, providing a yield of 0.43 g/g glucose and a productivity of 0.48 g/h·L, respectively. To the best of our knowledge, this is the highest titer ever reported using the NBS minimal medium. Moreover, to check the robustness of the bidirectional switch, the strain S8 was evaluated using the enriched medium under the same optimized conditions. After 72 h of fed-batch fermentation, a final OD_600_ of 45 could be detected in the enriched medium, which was 2.4-fold compared to minimal medium (OD_600_ of 19). Meanwhile, a final shikimic acid titer of 76 g/L and 1.05 g/h·L overall productivity could be obtained with strain S8 using the enriched medium (Fig. 4f). Notably, the average shikimic acid productivity (1.49 g/h·L) after 24 h was 6.8 times higher than the average productivity (0.19 g/h·L) before 24 h, indicating that the optogenetic switch could effectively control the carbon flux distribution based on light signal input.

## Discussion

Described herein, two optogenetic systems, TPAS and TPRS, were created by reconstructing the native TetR and TEVp using protein split technology. Besides, a light-controlled switch was merged from both systems to exhibit bifunctional regulation at the gene transcription and protein levels. Ultimately, the bifunctional switch successfully decoupled growth and production phases, raising shikimic acid titer to the highest ever recorded in *E. coli* in a minimal medium without adding costly aromatic amino acids.

The TPRS and TPAS systems enabled quick control of gene expression and protein degradation, respectively. To date, reported regulatory tools mainly use the gene transcription regulation, which is less ATP-driven while exhibiting easy controllability. However, transcription-based regulation tools are currently replaced by the protein control level [28] due to their quick control over cellular functions [29]. The present tools present integrated transcription and protein regulation tools, hereby exhibiting two main advantages: (i) Expensive inducers were avoided to decrease cytotoxicity and system crosstalk. Recent research highlighted the circuits manipulating protein expression [30, 31] and the tRNA mediated biosensor circuits [32] with efficient activation; however, they are highly inducer-dependent [33]. Oppositely, the use of light as the input signal moderates the cost of the fermentation process along with the system controllability [34, 35]; (ii) With the help of computer-based modelling and split energy analysis tools, a tunable platform of eight activatable TetR split pairs was constructed. Compared to other systems that provide a single tool alternative [36], different split pairs were designed to exhibit a wide dynamic range from 1.75- to 12.4-fold. This strategy could help to reprogram other proteins towards desired functions and controllability.

The bifunctional optogenetic switch, which also exhibited good composability, enabled a two levels control regulating gene transcription and protein accumulation. Currently, developing switches that combine both levels of regulations require good composability and orthogonality to ensure a predictable outcome [37]. Here, we present a strategy to circumvent the humps in mining new regulatory elements; specifically, two orthogonal proteins, TetR and TEVp, were designed and optimized to control the transcriptional and protein level, respectively. To date, the regulatory switches available have been exploited for upregulation [38], downregulation, or bifunctional regulation [39]. However, most switched designs operate on one regulatory level, such as transcriptional level [33, 40, 41] or protein level [42]. The transcription regulation switch demonstrated successful control, because it imposes little burden on cells, but its system control is slow [43]. Herein, protein regulation switches are regulated through protein degradation, giving a faster response and quick cellular function change. Moreover, this switch achieved bidirectional regulation by integrating resource-efficient transcriptional level regulation [44] with fast-responding protein level regulation [39].

Application of the bifunctional optogenetic switch to decouple cell growth and shikimic acid pathway resulted in the highest titer of shikimic acid reported while using the NBS minimal medium. Product yields in microbial cell factories are currently limited by metabolic flux competition between biomass production and biosynthesis. Therefore, several strategies to decouple growth and production phases have been created, including the application of substrate-dependent promoters [45], protein degradation based cell growth [46], CRISPRi governed intermittent gene expression [41], light controlled production phase [47], aptamer-based regulatory biosensor [48], etc. Herein, the potential of optogenetics in decoupling cell growth and shikimic acid production was demonstrated for the first time in shikimic acid production, and a more than twofold higher shikimic acid titer than previous reports was obtained under mineral salt medium. For the biosynthesis of shikimic acid, several static strategies were used to improve the titer of shikimic. For instance, two-stage fermentation [49], gene knockout [50], and key pathway gene optimization [51] were used to increase shikimic acid titer, producing 7.98, 13.1, and 101 g/L, respectively. Recently, dynamic regulation was used to rescue the use of expensive aromatic amino acids. For example, a CRISPRi based gene expression achieved 23 g/L shikimic acid, a 115% increase than the wild-type strain [52]. Moreover, several autonomous circuits, using the protease-based dynamic regulation circuit, growth phase-based dynamic regulation circuit, and QS-based dynamic regulation circuit, were constructed to boost the shikimic acid titer up to 12.63 g/L [53], 14.33 g/L [39], and 16.85 g/L [54], respectively. In the present study, we demonstrated the potential of the optogenetic switch to improve the shikimic acid titer up to 76 g/L under an enriched medium. Those results indicated that the present switch was robust and might provide valuable capabilities in metabolic engineering for valuable product biosynthesis. In fine, the present research and other previously published articles using optogenetic tools have been demonstrated to be effectively applied in 5 L fermenters to produce value-added chemicals [13, 55, 56]. The development of the adapted bioreactors and their respective light systems is crucial for deploying the light-controlled systems to the industrial level.

Despite the benefit of the TPRS and TPAS systems for regulating metabolic pathways, as discussed in the present work, these systems depend on multiple proteins built from cellular resources and may cause a metabolic burden to cells. As a result, novel and adaptive strategies can be applied to creating robust and much simpler systems that may reduce cellular burdens [57, 58]. Among those strategies, orthogonal DNA binding systems such as bZip, RNAi, or other synthetic DNA binding strategies to avoid non-specific recombination or protein aggregation before activation. In addition, a combination of different throughput strategies, such as omics analysis and high throughput screening, can help select the cells harboring the best tools with less metabolic burden. In terms of system high sensitivity, the present tool sensitivity can be expanded using different strategies such as computational and mathematical simulations to achieve a quick approach to select and predict new synthetic regulatory tools.

In summary, we demonstrated that chemically induced repressor protein and protease could be reprogrammed to develop robust regulatory circuits. Moreover, a bifunctional optogenetic switch with both regulatory functions achieved by multilevel controls was constructed to improve the shikimic acid to the highest titer ever reported in the minimal medium by *E. coli*.

## Methods

### Protein structure analysis and DNA assembly

Protein split sites for TetR were analyzed using the Pymol software and the computer-based protein split simulation SPELL [59]. A series of sub-cloning plasmids, including protein split fragments, vivid photodimers (VVD), and the dimerization control domain, were constructed using conventional cloning methods or Gibson isothermal assembly. Each protein split fragment was fused to a VVD fragment used as the dimerization control domain. Two sub-cloning modules were constructed and expressed in one plasmid. In addition, a glycine–serine-rich linker was used to connect the fragments to the dimerizing domain. The TEVp and TetR complete sequence prototype was used as the starting fragments for PCR amplification of fragments that included a 5' sequence to code for an *EcoRI restriction* site and a Kozak consensus sequence, as well as a 3' sequence to code for a *HindIII* restriction site, for the VVD fused N-terminal protein split fragments. The same method was used to amplify fragments that included a 5' sequence to code for a *HindIII restriction* site, a Kozak consensus sequence, and a 3' sequence to code for an XhoI restriction site with VVD fused C-terminal protein split fragments. PCR fragments were extracted and purified using agarose gel extraction (Epoch Life Science). The recombinant plasmids containing all fragments were transformed into chemically competent *E. coli* cells and selected on LB-agar plates with ampicillin and chloramphenicol under 37 °C static conditions.

### Plasmids and strains

The plasmids and strains used in this study are given in Table 1. The fundamental plasmids were pJ01 (GenBank ID: MK234843) and pTet-1 (GenBank ID: MK234848), from which the TetR was excised to make the expression plasmid pTet-2 of the reporter module. The reporter protein GFP was PCR amplified and cloned into a pTet-2 plasmid using a one-step cloning protocol (TAKARA, exanaseI kit) to create a reporter module. Rapid PCR site-directed mutagenesis was used to generate the gene *aroG*^*fbr*^ (encoding DAHP synthase) with a feedback-resistant D146N mutation. The gene *aroB*^*opt*^ (encoding DHQ synthase), a codon-optimized version, was produced by optimizing the first eight codons. The successful cloning was confirmed by colony PCR, restriction mapping, and direct nucleotide sequencing [53].

**Table 1:** Strains used in this study

Strains/Plasmids	Relevant characteristics	Source
Strains		
K1	*E. coli* MG1655 carrying pJ01-TetR	This study
TRU_(N26/C27)_	*E. coli* MG1655 carrying pJ01-TetR-26, P_tet_-GFP	This study
TRU_(N36/C37)_	*E. coli* MG1655 carrying pJ01-TetR-36, P_tet_-GFP	This study
TRU_(N46/C47)_	*E. coli* MG1655 carrying pJ01-TetR-46, P_tet_-GFP	This study
TRU _(N68/C69)_	*E. coli* MG1655 carrying pJ01-TetR-68, P_tet_-GFP	This study
TRU _(N99/C100)_	*E. coli* MG1655 carrying pJ01-TetR-99, P_tet_-GFP	This study
TRU_(N104/C105)_	*E. coli* MG1655 carrying pJ01-TetR-104, P_tet_-GFP	This study
TRU_(N124/C125)_	*E. coli* MG1655 carrying pJ01-TetR-124, P_tet_-GFP	This study
TRU_(N166/C167)_	*E. coli* MG1655 carrying pJ01-TetR-166, P_tet_-GFP	This study
TRU_(N167/C168)_	*E. coli* MG1655 carrying pJ01-TetR-167, P_tet_-GFP	This study
TRU_(N169/C170)_	*E. coli* MG1655 carrying pJ01-TetR-169, P_tet_-GFP	This study
TRU_(N179/C180)_	*E. coli* MG1655 carrying pJ01-TetR-179, P_tet_-GFP	This study
XN	*E. coli* MG1655, Δ*LacI*	This study
TAU_(N124/C125)_	XN carrying pJ01-TetR-124, P_tet_ LacI-GFP	This study
TAU_(N46/C47)_	XN carrying pJ01-TetR-46, P_tet_ LacI-GFP	This study
TAU_(N169/C170)_	XN carrying pJ01-TetR-169, P_tet_ LacI-GFP	This study
TAU_(N179/C180)_	XN carrying pJ01-TetR-179, P_tet_ LacI-GFP	This study
TAU_(N26/C27)_	XN carrying pJ01-TetR-26, P_tet_ LacI-GFP	This study
TAU_(N166/C167)_	XN carrying pJ01-TetR-166, P_tet_ LacI-GFP	This study
TAU_(N167/C168)_	XN carrying pJ01-TetR-167, P_tet_ LacI-GFP	This study
TAU_(N68/C69)_	XN carrying pJ01-TetR-68, P_tet_ LacI-GFP	This study
PRU-118	*E. coli* MG1655 carrying TEVp-118, P_tet_-(TeF)GFP	This study
PRU-118-NW	*E. coli* MG1655 carrying TEVp-118-NW, P_tet_-(TeF)GFP	This study
PAU-118	*E. coli* MG1655 carrying TEVp-118, P_tet_-GFP(LAA)	This study
PAU-118-NW	*E. coli* MG1655 carrying TEVp-118-NW, P_tet_-GFP(LAA)	This study
TPRS	*E. coli* MG1655 carrying pJ01-TetR_(166)_-TEVp_(118)_, P_tet_-(TeF)GFP	This study
TPAS	XN carrying pJ01-TetR_(166)_-TEVp_(118)_, P_tet_-(TeF)LacI-GFP(DAS)	This study
S4	*E. coli* MG1655, Δ*ptsHIcrr*::*Zmglf*, Δ*aroL*::*tktA*, Δ*aroK*, P_J23119_*-*GB	Lab stock
S5	S4, Δ*aroK*::*rpE*, Δ*yidB*	This study
S6	S5 carrying pJ01-TetR_(166)_-TEVp_(118)_, NX1	This study
S7	S5 carrying pJ01-TetR_(166)_-TEVp_(118)_, NX2	This study
S8	S5 carrying pJ01-TetR_(166)_-TEVp_(118)_, NX3	This study
Plasmids		
pJ01	P_J23119_ promoter, pMB1 ori, Amp^R^	Lab stock
pJ01-TetR-26	pJ01-containing B0034RBS, TetR_(N26)_-VVD, VVD-TetR_(C27)_	This study
pJ01-TetR-36	pJ01-containing B0034RBS, TetR_(N36)_-VVD, VVD-TetR_(C37)_	This study
pJ01-TetR-46	pJ01-containing B0034RBS, TetR_(N46)_-VVD, VVD-TetR_(C47)_	This study
pJ01-TetR-68	pJ01-containing B0034RBS, TetR_(N68)_-VVD, VVD-TetR_(C69)_	This study
pJ01-TetR-99	pJ01-containing B0034RBS, TetR_(N99)_-VVD, VVD-TetR_(C100)_	This study
pJ01-TetR-102	pJ01-containing B0034RBS, TetR_(N104)_-VVD, VVD-TetR_C105)_	This study
pJ01-TetR-124	pJ01-containing B0034RBS, TetR_(N124)_-VVD, VVD-TetR_(C125)_	This study
pJ01-TetR-166	pJ01-containing B0034RBS, TetR_(N166)_-VVD, VVD-TetR_(C167)_	This study
pJ01-TetR-167	pJ01-containing B0034RBS, TetR_(N167)_-VVD, VVD-TetR_(C168)_	This study
pJ01-TetR-169	pJ01-containing B0034RBS, TetR_(N169)_-VVD, VVD-TetR_(C170)_	This study
pJ01-TetR-179	pJ01-containing B0034RBS, TetR_(N179)_-VVD, VVD-TetR_(C180)_	This study
pJ01-TetR	pJ01-containing B0034RBS, TetR_(Full length)_	This study
PTet-1	P_tet,_ p15A ori, Cm^R^, TetR	Lab stock
PTet-2	P_tet,_ p15A ori, Cm^R^	This study
P_tet_-GFP	PTet-2 containing B0034RBS, GFP	This study
P_tet_ LacI-GFP	PTet-2 containing P_tet_, B0034RBS, LacI, P_trc_, B0034RBS, GFP	This study
TEVp-118	pJ01 containing P_j23119_, B0034RBS, TEVp_(N118)_-VVD, VVD-TEVp_(C180)_	This study
TEVp-118-NW	pJ01 containing P_j23119_, B0034RBS, VVD-TEVp_(N118)_, VVD-TEVp_(C180)_	This study
P_tet_-(TeF)GFP	PTet-2 containing P_tet_, B0034RBS, (TeF)GFP	This study
P_tet_-GFP(LAA)	PTet-2 containing P_tet_, B0034RBS, GFP(LAA)	This study
pJ01-TetR_(166)_-TEVp_(118)_	pJ01 containing B0034RBS, TetR_(N166)_-VVD, VVD-TetR_(C167)_, P_j23119_-VVD-TEVp_(N118)_, VVD-TEVp_(C180)_	This study
P_tet_-(TeF)LacI-GFP(DAS)	PTet-2 containing P_tet_, B0034RBS, (TeF)LacI, GFP fused with DAS tag	This study
pJ01-TEVp	pJ01 containing P_j23119_, B0034RBS, TEVp_(Full length)_	This study
PJ01*-*GB	pJ01 containing P_j23119_, B0034RBS, AroG, AroB	This study
NX-1	PTet-2 containing P_tet_, B0034RBS, (TeF)AroK	This study
NX-2	PTet-2 containing P_tet_, B0034RBS, (TeF)LacI, P_trc_, B0034RBS, AroG(DAS), AroB(DAS)	This study
NX-3	PTet-2 containing P_tet_, B0034RBS, (TeF)AroK, (TeF)LacI, P_trc_, B0034RBS, AroG(DAS), AroB(DAS)	This study

To create chassis strains for shikimic acid synthesis, chromosomal genes, such as *ydiB* (encoding quinate/shikimate dehydrogenase) and *aroK* (encoding shikimate kinase I) were knocked out in *E. coli* MG1655 using the CRISPR/Cas9 technique [53]. The *aroL* gene was replaced in the genome with a *tktA* expression cassette controlled by the J23119 promoter and the B0034 RBS. The PTS system (*ptsH, ptsI,* and *crr*, which encode *HPr,* EI, and EIIAGlc, respectively) was replaced in the genome with a glucose facilitator protein gene, *Zmglf*, from *Zymomonas mobilis.*

### Culture conditions

For genetic experiments, strains were cultured in LB broth containing 10 g/L tryptone, 5 g/L yeast extract, and 10 g/L NaCl. Ampicillin (100 mg/L) and chloramphenicol (30 mg/L) were added for plasmid maintenance. Shake flask fermentation for shikimic acid production was carried out in NBS medium.

NBS inorganic salt medium contains: glucose 20 g/L, KH_2_PO_4_ 3.5 g/L, K_2_HPO_4_ 5.0 g/L, (NH_4_)_2_HPO_4_ 3.5 g/L, CaCl_2_·2H_2_O 15 mg/L, trace element solution 0.67 mL/L. After the medium is sterilized, added with MgSO_4_·7H_2_O 0.25 g/L, V_B1_ 0.5 mg/L, and betaine hydrochloride 1 mM. Trace element in liquid composition: FeCl_3_·6H_2_O 2.4 g/L, CoCl_2_·6H_2_O 0.3 g/L, CuCl_2_ 0.15 g/L, ZnCl_2_·4H_2_O 0.3 g/L, NaMnO_4_ 0.3 g/L, H_3_BO_3_ 0.075 g/L, MnCl_2_·4H_2_O 0.5 g/L, dissolved in 0.1 M HCl.

The enriched fermentation medium contained: glucose 10 g/L, yeast extract 18 g/L, KH_2_PO_4_ 4.0 g/L, Na_2_HPO_4_ 1.0 g/L, (NH_4_)_2_SO_4_ 2.0 g/L, citric acid 2.0 g/L, MgSO_4_ 0.4 g/L, NaOH 3.0 g/L, and a trace element solution of 1 mL/L. Trace element liquid composition: FeSO_4_·7H_2_O 2.8 g/L, CoCl_2_·6H_2_O 2.5 g/L, CuSO_4_·5H_2_0 0.3 g/L, ZnSO_4_·7H_2_O 0.3 g/L, NaMoO_4_·2H_2_O 2.0 g/L, H_3_BO_3_ 1.0 g/L, MnSO_4_·H_2_O 1.7 g/L, CaCl_2_·2H_2_O 1.13 g/L, dissolved in 0.1 M HCl.

### Measurement of fluorescence intensity

The performance of different systems and bifunctional switch were evaluated. Cells of *E. coli* JM109, containing the respective plasmids, grew overnight at 200 rpm in 20 mL of LB with an appropriate concentration of antibiotics at 37 °C. Using the overnight culture, a new flask of 50 mL LB medium was inoculated to a diluted cell culture of 1:100 supplemented with antibiotics and grown for 2 h before analysis. The control samples were treated in similar conditions, and each sample fluorescence was analyzed and recorded. The assessment was carried out using a spectramax M3 microplate reader (Molecular Devices, USA). The excitation and emission wavelengths for GFP were at 488 ± 10 nm and 510 ± 10 nm, respectively. The experiment was taken in triplicates.

### Flow cytometry measurements

During time-course studies, samples were collected from cell cultures, washed twice with PBS (pH = 7.2), and re-suspended to an OD_600_ of 0.2. A BD Biosciences LSR Fortessa instrument was utilized during the studies, outfitted with fluorescein isothiocyanate (FITC) (GFP) channels. To compensate, cells that exclusively transmit GFP were employed. At a flow rate of 0.5 mL/s, each sample received at least 10, 000 counts. All data exported in FCS3 format were processed using the Flow Jo software (FlowJo, LLC).

### Kinetic parameter assay

To further validate the advantages of utilizing the combined regulatory tools, kinetic parameters were evaluated using time Lapse fluorescence measurements across cells carrying the same regulatory tool under light conditions, which were subsequently fitted to prism pad statistical analysis.

Cells containing TPRS and its modular components (TRU and PRU) were grown overnight for the protein depletion regulation tools. On day 2, they were moved to a fresh 50 mL shake flask with a 500 µL inoculation volume and incubated for 2 h before turning on the light, and every 30 min for 3 h, a sample of 1 mL of culture was collected for fluorescence measurement. The control was grown in identical conditions but under a light-deprived environment (dark).

Cells carrying TPAS and its modular components (TAU and PAU) were grown overnight for the protein accumulation regulatory tools. On day 2, they were moved to a fresh 50 mL shake flask with a 500 µL inoculation volume, and then the light was turned on to promote protein accumulation. *t* = 0 was then recorded, and samples were collected every 30 min for 3 h for fluorescence examination.

### Fluorescence microscopy assay

Bacterial cells were collected by centrifugation at 1500 rpm for 10 min, followed by two washes with PBS (pH 7.2). Microscopy images were taken using a Nikon ECLIPSE 80i microscope equipped with a Nikon DS-Ri1 camera.

### Shikimic acid fermentation

The seeds were grown in LB medium overnight at 37 °C and 200 rpm. Then, with the starting bacterial concentration controlled at OD600 = 0.05, transfer to NBS minimal medium or enriched medium containing 20 g/L glucose, 100 mg/L ampicillin, and 30 mg/L chloramphenicol. A 250 mL shake flask with a working capacity of 50 mL serves as the fermentation system. The culture temperature was kept at 37 °C, and the rotation speed was 200 rpm. Experiments on batch and fed-batch shikimic acid fermentation lasted for 72 h.

LED blue panels (450 nm, 22 W, 24 V, 1.7 A; MODEL: HF-FX160, square light source), purchased from KOMA Vision Technology and LEMONS Co., Ltd (China), were used to illustrate optogenetics. The lighting equipment was powered by an AC/DC ADAPTER (MODEL: SPF-1210) (AC 100–240 V 50/60 Hz; OUTPUT: DC 12 V, 10 A). To improve the applicability and stability of scale, four blue light sources were used to surround a 5 L fermenter, respectively. These light panels were set 5 cm away from the vessel sides. The culture pH, airflow, and temperature were maintained at 6.0, 1 vvm, and 37 °C in a 5 L fermenter (INFORS, Switzerland). Every 6 h, samples were collected to test OD_600_ and DCW.

### Analytical methods

Shikimic acid concentrations in the fermentation medium were determined using a high-performance liquid chromatography system (Dionex UltiMate 3000 series; Thermo Scientific, MA, USA) equipped with an Atlantis® C_18_ column (5 m, 4.6 × 250 mm), eluted with 0.5 mM H_2_SO_4_ at a constant flow rate of 0.6 mL/min (55 °C), and detected by monitoring absorbance at 210 nm. A spectrophotometer was used to monitor cell growth through the OD_600_. All the experiments done were conducted in triplicates for statistical analysis. Differences between the two groups were determined by two-tailed student’s *t* test and paired sample analysis through SPSS statistics software (SPSS V13.0).

## Supplementary Information

**Additional file 1: Fig. S1.** TetR split sites and split-site selection. **Fig. S2.** Evaluation of the effects of genomic LacI on the transcription activation regulation tool. **Fig. S3.** Evaluation of light effects on cell growth. **Fig. S4.** Evaluation of the transcription unit performance. **Fig. S5.** TEVp cartoon model of the split-site location. **Fig. S6.** Tunability of the protein regulation unit. **Fig. S7.** Evaluation of the effects of protein level regulatory unit on cell growth**. Fig. S8.** Evaluation of the protein regulation unit. **Fig. S9.** Kinetics of the TPRS and TPAS. **Fig. S10.** Evaluation of cells harboring the transcription and proteolysis regulation systems. **Fig. S11.** Evaluation of the optimum temperature for cell growth and shikimic acid production. **Fig. S12.** Evaluation of the best initial glucose concentration for shikimic acid production. **Fig. S13.** Optimization of light switch time and shikimic acid production.

## Data Availability

All data generated or analyzed during this study are included in this published article and its additional files.

## Abbreviations

TRU: Transcription repression unit
TAU: Transcription activation unit
PRU: Protein accumulation repression unit
PAU: Protein accumulation activation unit
TPRS: Transcription and protein repression system
TPAS: Transcription and protein activation system
DCW: Dry cell weight
SA: Shikimic acid
QA: Quinic acid
