# Supplementary material for: Bifunctional optogenetic switch for improving shikimic acid production in E. coli
Source: Biotechnol Biofuels Bioprod. 2022 Feb 7;15:13. doi: 10.1186/s13068-022-02111-3 (PMC8822657; doi:10.1186/s13068-022-02111-3)
Supplement: Supplementary file 1 — Additional file 1: Fig. S1. TetR split sites and split-site selection. Fig. S2. Evaluation of the effects of genomic LacI on the transcription activation regulation tool. Fig. S3. Evaluation of light effects on cell growth. Fig. S4. Evaluation of the transcription unit performance. Fig. S5. TEVp cartoon model of the split-site location. Fig. S6. Tunability of the protein regulation unit. Fig. S7. Evaluation of the effects of protein level regulatory unit on cell growth. Fig. S8. Evaluation of the protein regulation unit. Fig. S9. Kinetics of the TPRS and TPAS. Fig. S10. Evaluation of cells harboring the transcription and proteolysis regulation systems. Fig. S11. Evaluation of the optimum temperature for cell growth and shikimic acid production. Fig. S12. Evaluation of the best initial glucose concentration for shikimic acid production. Fig. S13. Optimization of light switch time and shikimic acid production. [file 13068_2022_2111_MOESM1_ESM.docx]

Additional file 1

**Bifunctional optogenetic switch for improving shikimic acid production in *E. coli***

Irene Komera ^a,b^, Cong Gao^a,b^, Liang Guo^a,b^, Guipeng Hu^c^, Xiulai Chen^a,b^, Liming Liu^a,b,*^

^a^State Key Laboratory of Food Science and Technology, Jiangnan University, Wuxi 214122, China;

^b^International Joint Laboratory on Food Safety, Jiangnan University, Wuxi 214122, China;

^c^School of Pharmaceutical Science, Jiangnan University, Wuxi 214122, China.

*Corresponding author e-mail: [mingll@jiangnan.edu.cn](mailto:mingll@jiangnan.edu.cn) (Liming Liu).

The supplementary materials file contains:

Additional file 1 Figures from 1 to 13.

Additional file 1 figures


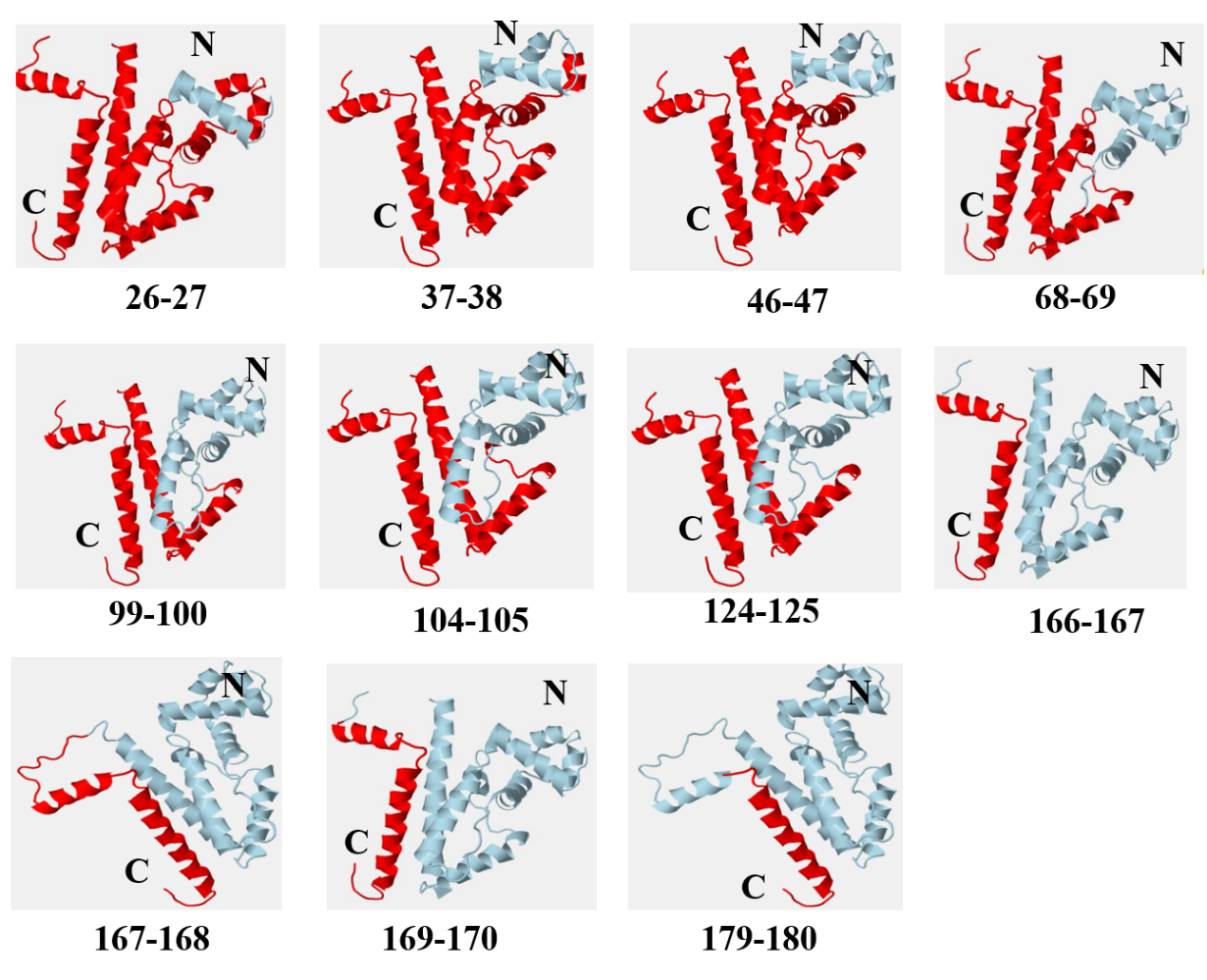


**Additional file 1 Fig. 1. TetR split sites and split-site selection.** The TetR cartoon model displays the split-site location and the catalytic residue. At the same time, the silver domain represents the N terminal of the split whereas, the red domain represents the C-terminal of the split. After PDB sequence alignment, the PDB template (2V2G), FASTA sequence was aligned with our TetR sequence for homology/site confirmation. The homology alignment, split energy profile, and the concerved residues were analyzed for each split-site.


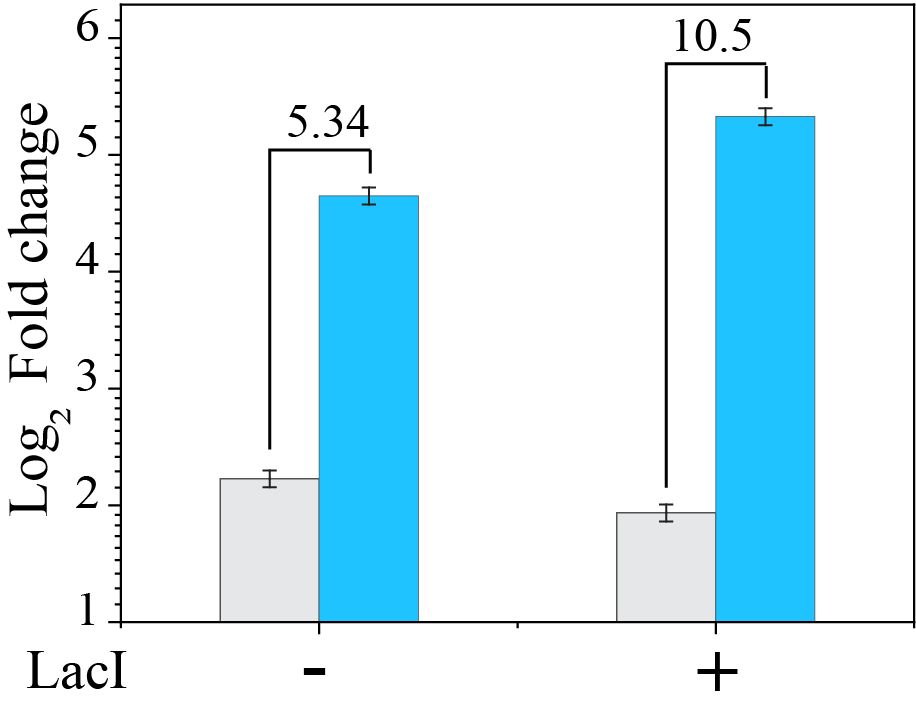


**Additional file 1 Fig. 2. Evaluation of the effects of genomic LacI on the transcription activation regulation tool.** The same TAU-116 plasmid was transformed in two strains; with (+) or without (-) genomic LacI. The samples were collected in triplicates.


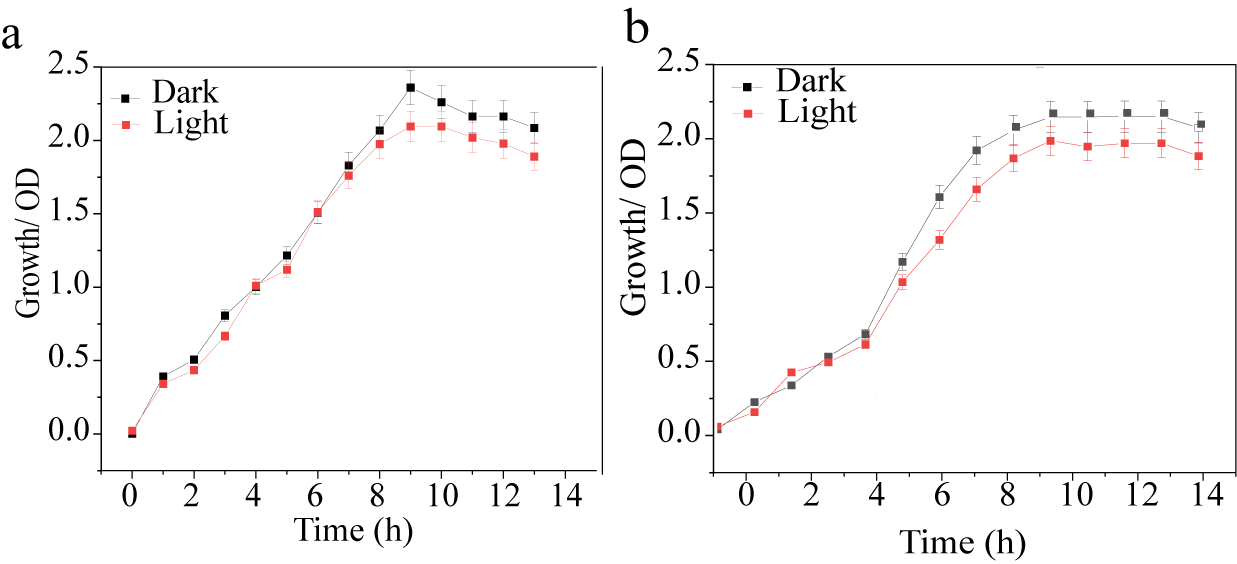


**Additional file 1 Fig. 3. Evaluation of light effects on cell growth.** Cells harboring either the transcription repression or the transcription activation unit were analyzed by monitoring time-lapse cell growth. (**a**) Growth profile of the cell holding the transcription repression units. (**b**) The growth profile of the Tet-166 splits containing strain under the transcription activation unit.


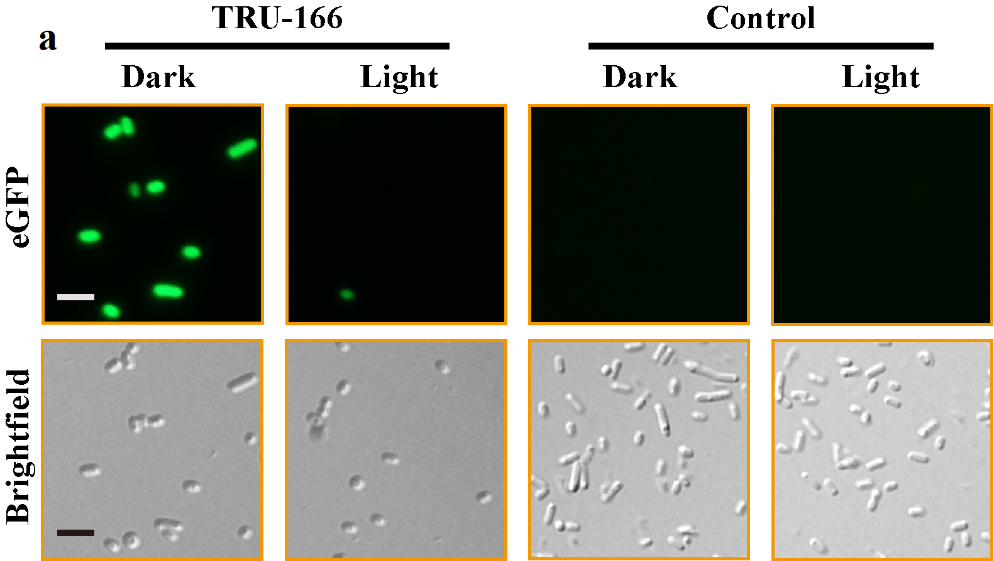

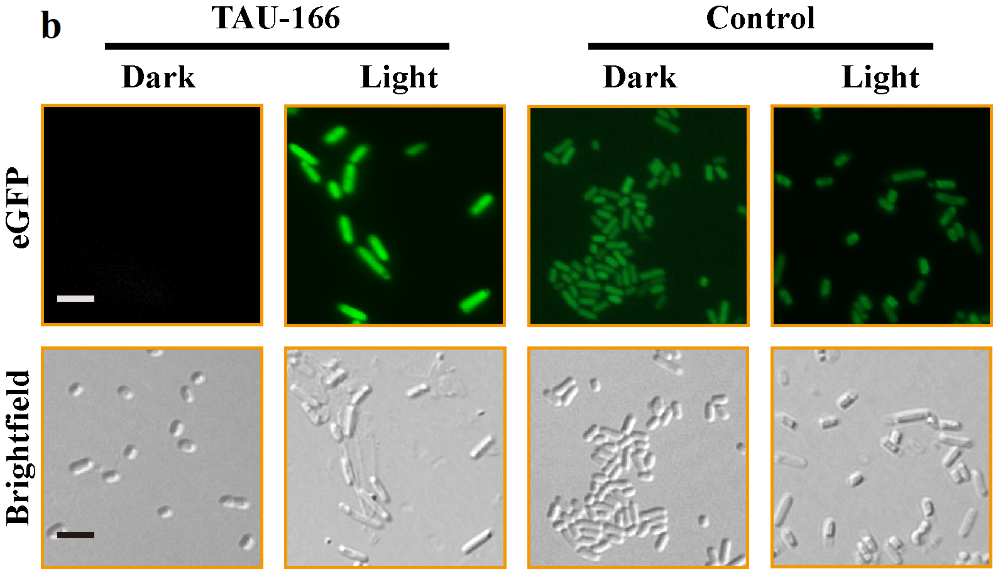


**Additional file 1 Fig. 4. Evaluation of the transcription unit performance.** (**a**) Single Cell fluorescence imaging of the166 split pair containing cells under the transcription repression unit. In dark and light conditions. (**b**) Cell fluorescence imaging of the166 split pair containing cells under the transcription activation unit treated with dark and blue light conditions for cell response records. The control strain expressed complete TetR protein constitutively.


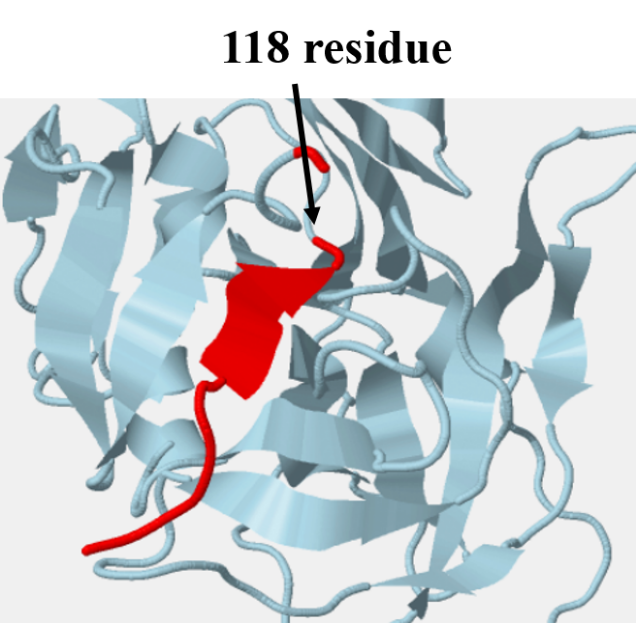


**Additional file 1 Fig. 5. TEVp cartoon model of the split-site location.** The silver domain represents the N terminal of the split, whereas the red domain represents the C-terminal of the split.

**
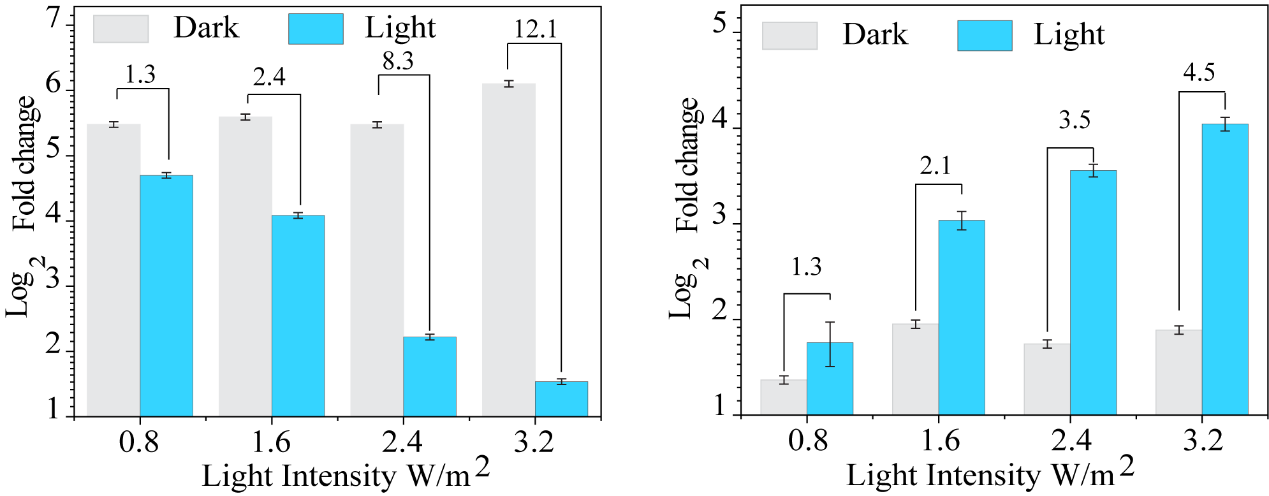
**

**Additional file 1 Fig. 6. Tunability of the protein regulation unit.** The tunability of the protein regulation unit was investigated through fluorescence accumulation or depletion; the light intensity was used as the control knob. (**a**) Tunability of the protein accumulation depletion unit. (**b**) Tunability of the protein accumulation activation unit.


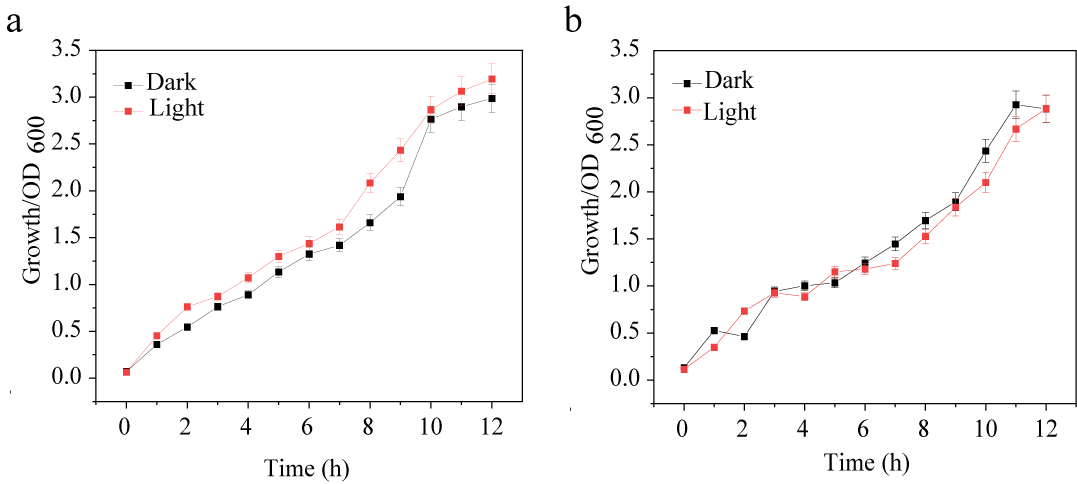


**Additional file 1 Fig. 7. Evaluation of the effects of protein level regulatory unit on cell growth**. (**a**) Growth profile of the cell harboring the protein accumulation repression units. (**b**) The growth profile of the strain containing the protein accumulation activation unit.


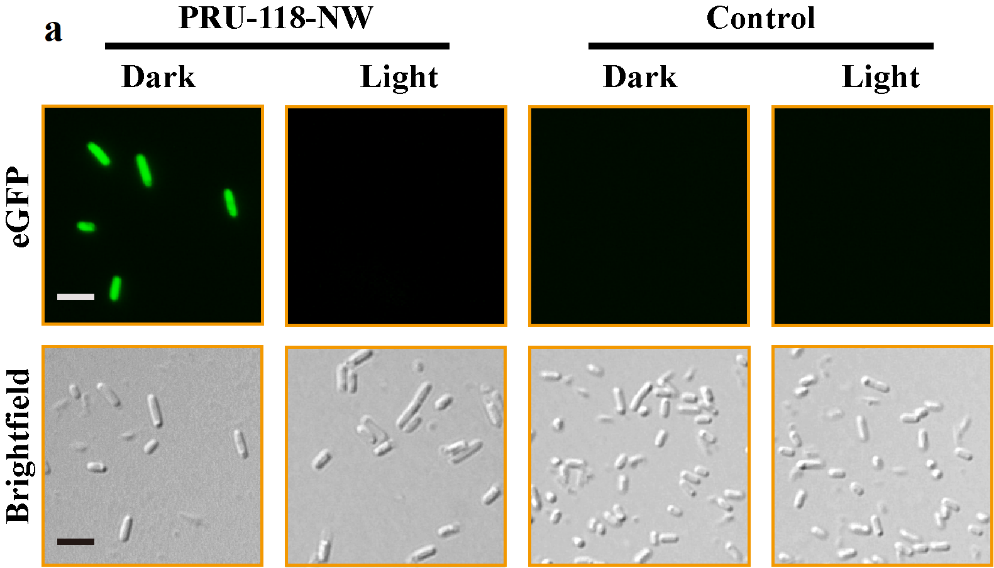

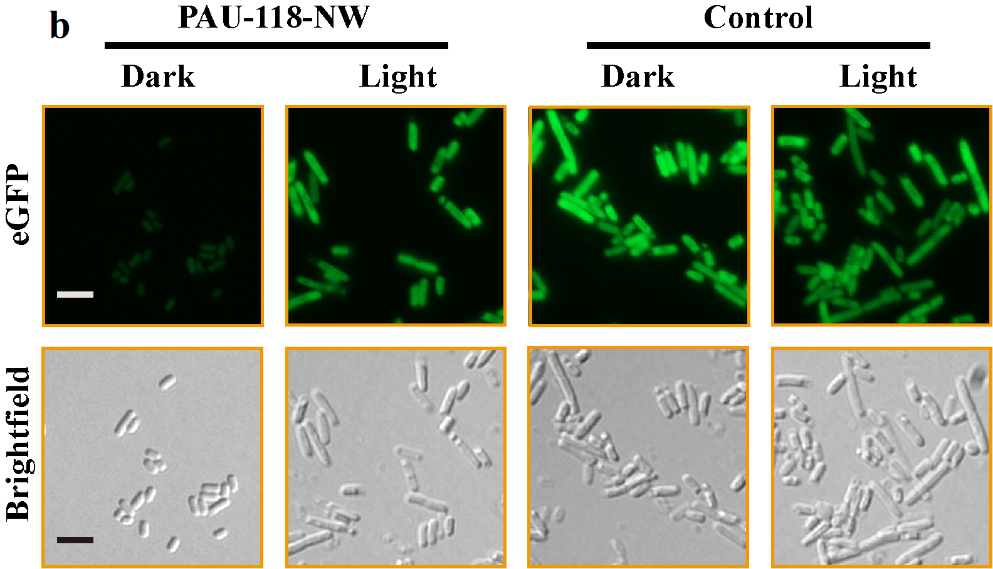


**Additional file 1 Fig. 8. Evaluation of the protein regulation unit.** (**a**) Single-cell fluorescence imaging of the TEVp-118 split pair containing cells under the protein accumulation repression unit. (**b**) Cell fluorescence imaging of the TEVp-118 split pair containing cells under the protein accumulation activation unit. The control strain expressed complete TEVp protein constitutively.


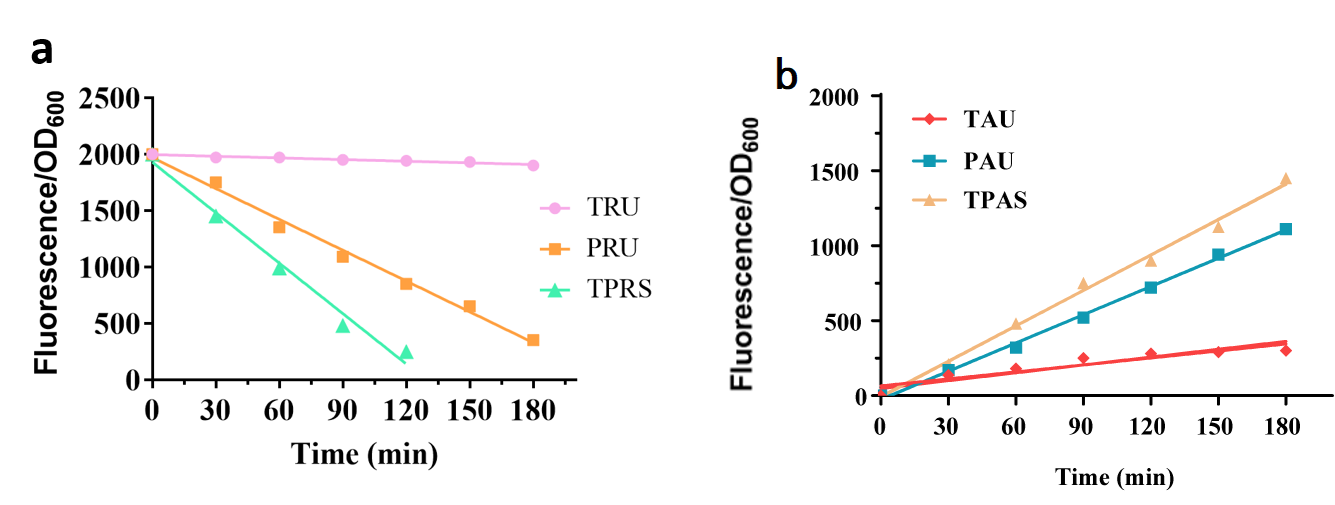


**Additional file 1 Fig. 9. Kinetics of the TPRS and TPAS.** (**a**) The kinetics of the GFP depletion under TRU, PRU TPRS. (**b**) The kinetics of GFP accumulation under the TAU, PAU, and TPAS regulatory tools.


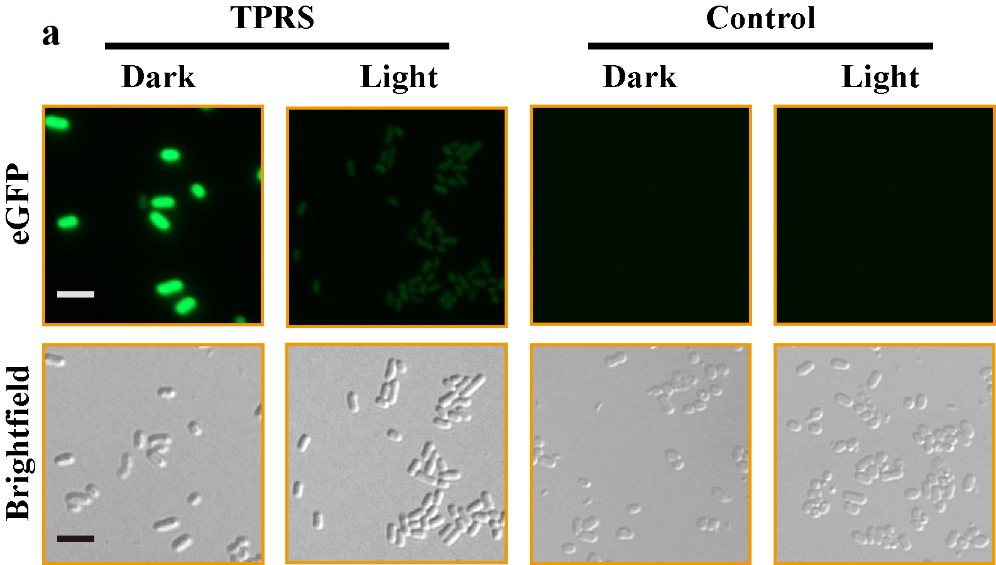


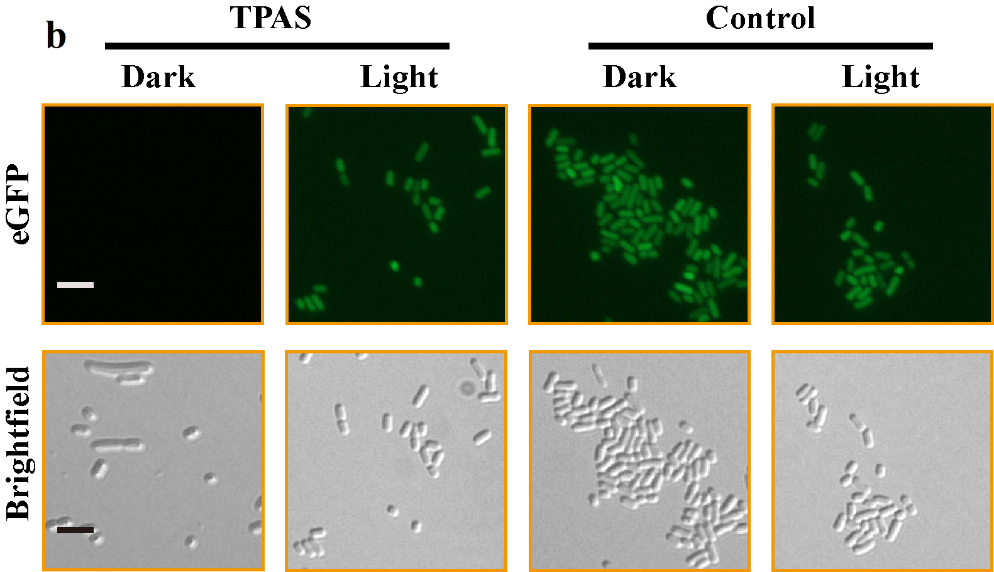


**Additional file 1 Fig. 10.** **Evaluation of cells harboring the transcription and proteolysis regulation systems.** (**a**) Single-cell imaging monitors the TPRS at the cellular level under dark and light conditions. (**b**) Fluorescence microscopy of the cells harboring the TPAS towards the dark and light-induced conditions. The control strain expressed complete tetR and TEVp proteins constitutively.


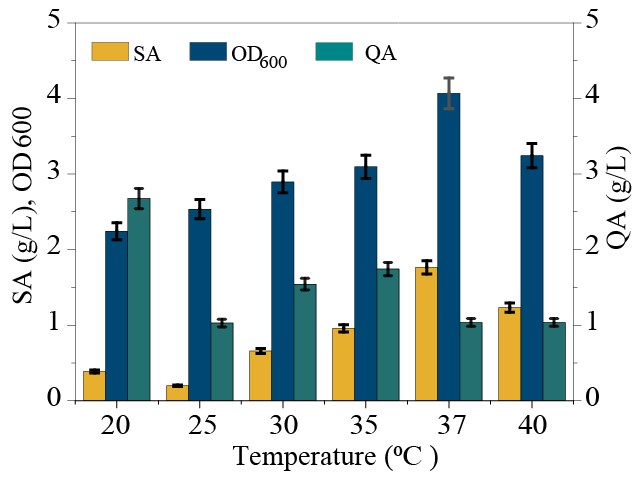


**Additional file 1 Fig. 11. Evaluation of the optimum temperature for cell growth and shikimic acid production.** To optimize biotransformation in shake flasks of cells harbouring the bifunctional switch, the temperature was the first condition optimized using 20 g/L glucose, and the temperature range investigated was between 20-40^o^C. After 72 h of fermentation, shikimic acid (SA), cell optical density (OD_600_), and quinic acid (QA) were also analyzed. The samples were taken in three biological replicates.


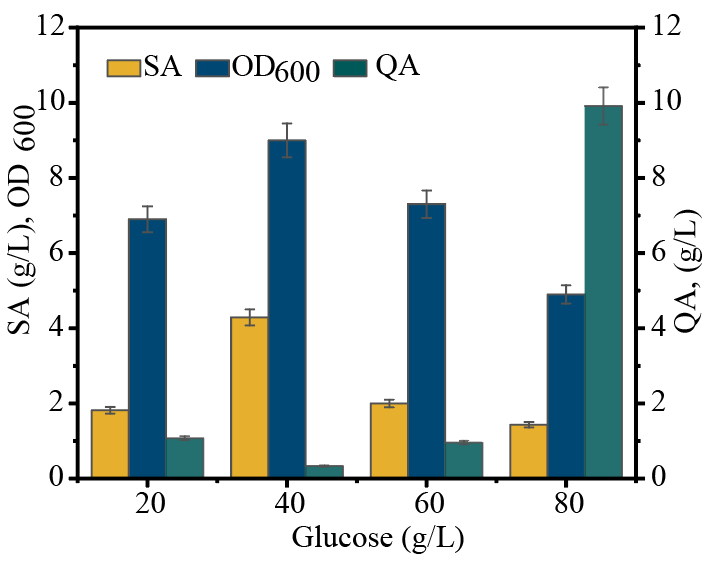


**Additional file 1 Fig. 12. Evaluation of the best initial glucose concentration for shikimic acid production.** The optimum concentration was investigated in the glucose concentration between 20 and 80 g/L under the optimum temperature 37^o^C. The appropriate concentrations were added at the time of inoculation. Values of shikimic acid (SA), cell optical density (OD_600_), and quinic acid (QA) are shown as mean ± s.d. from three biological replicate experiments.


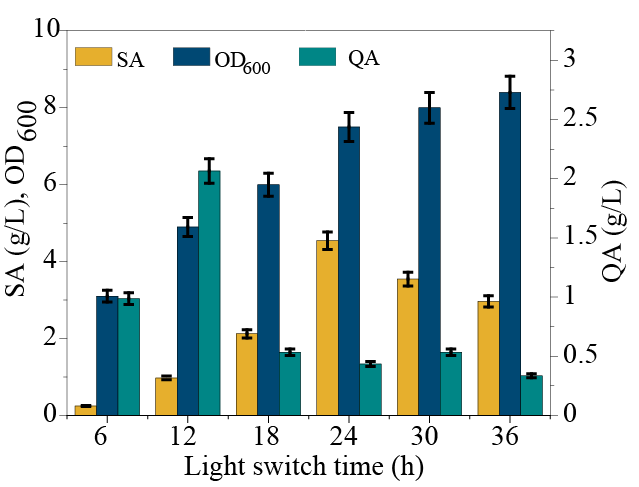


**Additional file 1 Fig. 13. Optimization of light switch time and shikimic acid production.** To determine the best time to shift from growth to shikimic acid production, we investigated the light switch time at various time points (6 h to 36 h) of cell growth using 40 g/L glucose at 37^o^C. Values of shikimic acid (SA), cell optical density (OD_600_), and quinic acid (QA) are shown as mean ± s.d. from three biological replicate experiments.
